# Cost-Effectiveness of Antiretroviral Therapy for Prevention

**DOI:** 10.2174/157016211798038542

**Published:** 2011-09

**Authors:** James G Kahn, Elliot A Marseille, Rod Bennett, Brian G Williams, Reuben Granich

**Affiliations:** 1 University of California, San Francisco, USA;; 2 Health Strategies International, Oakland, CA, USA;; 3 Independent Consultant, UK;; 4 South African Centre for Epidemiological Modelling and Analysis, Stellenbosch, South Africa;; 5 World Health Organization, Geneva, Switzerland

**Keywords:** Antiretroviral therapy, cost analysis, cost-effectiveness, economics, HIV prevention.

## Abstract

Recent empirical studies and analyses have heightened interest in the use of expanded antiretroviral therapy (ART) for prevention of HIV transmission. However, ART is expensive, approximately $600 per person per year, raising issues of the cost and cost-effectiveness of ambitious ART expansion. The goal of this review is to equip the reader with the conceptual tools and substantive background needed to understand and evaluate the policy and programmatic implications of cost-effectiveness assessments of ART for prevention. We provide this review in six sections. We start by introducing and explaining basic concepts of health economics as they relate to this issue, including resources, costs, health metrics (such as Disability-Adjusted Life Years), and different types of economic analysis. We then review research on the cost and cost-effectiveness of ART as treatment, and on the cost-effectiveness of traditional HIV prevention. We describe critical issues in the epidemic impact of ART, such as suppression of transmission and the role of the acute phase of infection. We then present a conceptual model for conducting and interpreting cost-effectiveness analyses of ART as prevention, and review the existing preliminary estimates in this area. We end with a discussion of future directions for programmatic demonstrations and evaluation.

## INTRODUCTION

Over the past three years, diverse developments in global Human Immunodeficiency Virus (HIV) control have fostered greatly increased interest in the use of expanded antiretroviral therapy (ART) for prevention of HIV transmission, and particularly in the cost and cost-effectiveness of that strategy. Perhaps foremost has been frustration with the inability of traditional HIV prevention strategies, such as condom distribution and individual-level behavioural interventions, to control the generalised epidemic in many settings [1]. In parallel, several factors have made broad ART use easier to contemplate: simpler-to-take pill regimens, lower antiretroviral drug prices (as little as United States [US]$169 per person per year for the World Health Organization [WHO]’s recommended first line of tenofovir/lamivudine/efavirenz [2]), and a rapid expansion of the number of individuals on ART to 5.25 million by the end of 2009 [3]. New treatment guidance from WHO in 2010 and reflected in the subsequent *Treatment 2.0 *initiative, expands the indication for ART to ≤350 CD4 cells/mm^3 ^[4]. Further, accumulating evidence suggests that ART suppression of viral load reduces HIV transmission by about 92% [5, 6]. The *HIV Prevention Trials Network (HPTN) 052* randomised clinical trial was stopped four years early when the Data Safety Monitoring Board observed a 96% reduction in transmission in those who started ART immediately when below CD4 count of 550 cells/mm^3 ^versus those that waited [7]. Epidemic modelling suggests very substantial benefits from well-done ART expansion [8]. The potential for and potential gains from expanded ART have never been clearer.

However, real-world economics imposes practical constraints. Despite the millions of individuals receiving ART, there are over 9 million clinically-eligible individuals that lack access, even under older and more stringent ART eligibility standards [3]. Global health funding, such as the US President’s Emergency Plan for AIDS Relief (PEPFAR) programme, has stopped growing, with increased attention to alternate priorities for limited global health resources [9]. Health system capacity, rather than antiretroviral drug prices, may be the biggest short-term barrier to further expansion. In the longer term, drug prices may again ascend in importance, with the increasing reliance over time on more expensive second- and third-line therapies.

Thus, the increased appeal of expanded ART is advisedly considered in the context of relevent economics. Hence this review.

Our goal is to equip the reader with the conceptual tools to understand and evaluate the policy and programmatic implications of cost-effectiveness assessments of ART for prevention. To accomplish this, we address the following topics:

- Basic concepts of health economics as they relate to this issue, including resources, costs, health metrics (disability-adjusted life years [DALYs]), and cost-effectiveness (cost per DALY averted).
- ART as treatment costs and cost-effectiveness.
- Cost-effectiveness of traditional HIV prevention.
- Epidemic impact of ART as prevention, which determines the DALYs averted *via *reduced transmission.
- A conceptual model for conducting and interpreting these cost-effectiveness analyses, including which input data are needed and are likely to have substantial effects on costs, effectiveness, and cost-effectiveness.
- ART as prevention preliminary estimates of cost-effectiveness.
- Future directions, for programmatic demonstrations and evaluation.

## BASIC CONCEPTS IN HEALTH ECONOMICS

In this section we review economics terms and ideas that are central to understanding the issue of cost-effectiveness of ART. Table **2** provides a road map of the key types of cost analysis in health. These include descriptive methods (e.g. cost estimation), as well as analytic techniques designed to produce a measure of the efficiency of health interventions and programmes (e.g. cost-effectiveness).

The most basic economic measure is the ***cost*** of health care interventions or services. The cost represents the mix and number of input resources required to deliver these services, with attached costs. Specifically, intervention cost is the sum of the product of resources required to implement the intervention and their unit costs. The resources typically include personnel, supplies (consumables, e.g. medications and condoms), equipment, services (such as advertising or electricity), training, and facility space (e.g. rent). Each resource has a unit cost, such as the hourly wage for a nurse or the cost of a single test kit. When all resources are tallied and unit costs assigned, the sum is the cost. Most cost analyses evaluate “economic” costs, which often differ from financial flows. Economic costs represent the true value to society of those resources, regardless of what the programme actually paid. Thus, donated resources (e.g. volunteer time, test kits provided *gratis* from the government) would be valued at fair market value. The goal is to quantify true costs - the value of resources consumed - not the monetary transactions which depend on the idiosyncrasies of how organisations obtain resources from collaborating or funding agencies. “Financial” costs are useful for understanding short-term budgetary implications and are sometimes also reported. They represent what the implementing agency paid, regardless of true societal value.

*** Net cost*** reflects the cost of delivering services (as above), adjusted for offsetting savings due to disease averted. For example, starting ART is likely to decrease opportunistic infections and other medical problems, thus averting some future health care costs. Similarly, HIV infections averted by prevention reduce the net costs of that prevention programme by obviating the health care requirements of those who would otherwise have become ill. (Diagnostic tests may *induce* health care costs, which would add to net costs.) Offsetting savings can be greater than programme costs, in which case there are net savings (and no need to calculate a cost-effectiveness ratio; see below).

*** Cost-effectiveness analysis (CEA)*** is a technique that has been widely used in health care for several decades. A CEA compares the net cost with the units of health benefit gained, expressed in ratio form, such as the net cost per death averted. This ratio is called the “Incremental Cost-effectiveness Ratio” (ICER), because both the costs and health benefits are incremental - i.e. the added cost and added health benefits versus a less expensive and less effective intervention approach (or no intervention).

There is a specific type of CEA that has in recent years been widely accepted as the standard approach for health. The net cost component of the ratio, in the numerator, is the same as above. However, the health benefits in the denominator are translated into a standardised single metric of change in health, combining into one number both mortality and morbidity effects. In global health, this metric is “Disability Adjust Life Years” (DALYs) (Table **1**). DALYs are a measure of disease burden: the LY represents premature mortality, and the DA represents disability due to morbidity. Thus, if an individual loses two years of life due to illness, and also has a 20% disability compromise while alive, for five years, the DALY burden of that disease would be 2 + 0.2 * 5 = 3.0 DALYs. (If occurring over multiple years, DALYs are discounted to reflect time preferences, but that is beyond the scope of this review.) Thus interventions *avert* DALYs, and the ICER is the net cost per DALY averted.

“Quality-Adjusted Life Years” (QALYs) were developed before DALYs (Table **1**). They are still used for CEAs in the US and Europe. The QALY is a measure of health - essentially the negative of the DALY. Thus, an illness which shortens life by 2 years and lowers “health status utility” by 20% for 5 years would decrease QALYs by 3. Interventions are designed to increase QALYs, and the ICER is the net cost per QALY gained.

According to the World Health Organization, the attractiveness of the ICER can be determined by comparison with the country’s annual gross domestic product (GDP) *per capita*. An intervention with an ICER below the annual GDP *per capita* is considered “very cost-effective”. An ICER below three times the annual GDP *per capita* is considered “cost-effective” [10].

For any cost-effectiveness analysis, if there are net savings, the ICER is not used. This is because the ICER - intended to show the cost of health gains - has no intuitive meaning when costs drop as health rises. The ICER has no useful meaning when programmes result in net savings because there is no trade-off between costs and benefits. Instead, the convention is to report that the intervention is “dominant” - both cheaper and better, and to report the absolute magnitude of savings and health gains, with no ratio.

Some economists expand the cost component (the numerator) of CEA to reflect costs beyond medical care. They include “willingness to pay” for the health benefits, as an additional savings. This extends CEA into a realm traditionally left to cost-benefit analysis (see below), and is controversial because others consider it to duplicate the economic productivity element of the denominator (DALYs or QALYs).

*** Resource allocation*** is the assignment of available funds (e.g. a budget) to a mix of interventions to maximise a specified health goal (e.g., HIV infections or DALYs averted), subject to certain constraints, typically equity and political concerns. Thus, for example, HIV prevention funding might be allocated in part based on cost-effectiveness, and in part based on equity across groups and attention to different risk behaviours. The technically best solution (i.e. maximising health value for money) can be calculated based on a list (or “league table”) of ICERs, combined with information about how much each intervention can be scaled up (reflecting the availability of demand for services and of needed input resources, such as health care workers).

The league table is a listing of intervention options, from best to least performing (i.e. like a sports league list of team standings). It typically includes the name of the intervention, the programme cost for a specified number of individuals (e.g. 1000), the net cost, the health gain (in DALYs or QALYs), and the cost-effectiveness (CE) ratio compared with current standard of practice. The comparison may also be with a less intensive intervention (also in the table) addressing the same health problem, e.g. comparing an HIV prevention strategy for the entire adult population *versus* only those at high risk of HIV acquisition. Money would be allocated to each successively less cost-effective intervention until the budget was fully committed.

However, equity imperatives mean that in practice, budgets are allocated partly according to cost-effectiveness criteria, and partly to ensure that all important population groups (ethnicity, sex, geographic location, risk behaviours) are included. Political dynamics may also complicate the way funds are allocated and can produce funding outcomes that deviate substantially from health maximisation.

Equity and other non-economic factors can be incorporated into resource allocation in various ways. One is to attach an explicit cost to failure on non-economic standards, e.g. inequity, in the CE ratio. A strategy of reaching 1000 individuals in a particular group, to the exclusion of 1000 individuals in other population groups, may be deemed to “cost” society a certain amount - the value placed on equity. This cost is reflected in the numerator - the intervention net cost is increased accordingly. Alternatively, and more commonly, equity is addressed outside the league table. Interventions are characterised as less or more equitable. Choosing a more equitable strategy that is not as cost-effective is justified on non-economic criteria. Finally, this choice of equity (or any non-economic criterion) over efficiency can often be characterised in terms of its “shadow cost”. Shadow costs are the health or economic gains foregone by a decision based on other criteria. For example, if allocating $10 million in program funds, funding in part due to equity considerations may mean that 10 (or 50, or 100) HIV infections are *not* averted, as compared with the most efficient set of interventions. Or, $1 million (or $5 million, *etc.*) of economic savings are foregone.

*** Cost-benefit analysis (CBA)*** is a much less common type of analysis than CEA in health care. In CBA, the benefits that derive from improved health are monetised, rather than being expressed as DALYs or other health metrics. The CBA result is expressed, usually, as benefits minus costs. Thus, for example, the value of longer life and lower morbidity due to ART might be expressed as the sum of medical costs averted and economic productivity added, or in terms of the “willingness to pay” for ART of all affected individuals. This would be compared with the cost of delivering ART.

In CBA, all health outcomes including human life are assigned a dollar value. If benefits exceed costs, an activity is considered economically efficient. The power of CBA is that it permits comparisons of widely disparate funding alternatives. The value of spending on a new airport could be compared with spending on a maternal health initiative. However, the technical and philosophical underpinnings of the monetary valuation of health outcomes are controversial (even among some economists). The practical requirements of mustering the necessary data can also be onerous, especially for small agencies. For these reasons, CBA is rarely used in the economic assessment of health programmes.

CBA results are sometimes expressed as ratio of benefits to costs. This has the virtue of being unitless - the ratio is the same whether the intervention is 10% or 90% scaled up (assuming no economies or diseconomies of scale). In contrast, an arithmetic difference depends crucially on the scale selected. The disadvantage of a ratio is that it may be very sensitive to the placement of cost effects on the cost or benefit side. For example, with ART, are the reduced medical costs associated with decreased infections an adjustment to the cost, or a benefit? This somewhat arbitrary decision affects the final ratio.

CBA can answer the question, is it economically worthwhile to undertake the intervention? The advantage of CBA is that it can include a wider range of benefits not explicitly evident in an ICER, such as increased economic productivity (though the disability in DALYs and the utility in QALYs implicitly incorporate these). There are no standard rules regarding the kinds of benefits due to improved health to be counted (and monetised). CBA is much less common in health economics, due to its heterogeneous scope, lack of standardisation, de-emphasis of health outcomes, and, perhaps related, origin in welfare economics (whereas CEA grew from medical decision analysis).

## ART AS TREATMENT: COST AND COST-EFFECTIVENESS

ART is first and foremost a highly effective but relatively expensive treatment strategy. Until recently, cost-effectiveness analyses have focussed on the effects of ART administered at low CD4 cell counts (typically < 200 cells/mm^3^) in prolonging life and decreasing morbidity in the patients receiving the therapy, without considering prevention benefits. We are aware of five academic reports assessing the cost-effectiveness of ART in Africa (see below). Some CEAs compare ART with no intervention, though another appropriate comparison is with other effective and available care interventions, such as trime-thoprim-sulphamethoxazole (cotrimoxazole) prophylaxis.

One study in South Africa found that highly active antiretroviral therapy (HAART) was cost-saving for patients with AIDS due to savings in hospitalisation and other health expenditures, and cost US$675 per life-year gained for non-AIDS patients [11]. Another in South Africa found that HAART cost US$1631 per quality-adjusted life year (QALY) gained for all treated HIV patients [12]. A third study that ranked ART against other HIV prevention and treatment options in developing countries found an incremental cost ranging from $547 to $5175 (international dollars) per DALY averted [13]. The 10-fold range of results reflects varying order of intervention introduction, in which the sequence of prevention and ART addition affects benefits more than programme costs. The importance of this uncertainty depends in part on the standard for cost-effectiveness. As noted above, the WHO considers an ICER below annual gross domestic product (GDP) *per capita* to be “very cost-effective” and below three times this value as “cost-effective”. Depending on the setting, the uncertainty may not cross these definitional thresholds.

A study using a well-known simulation model predicted disease progression and treatment costs as a function of CD4 cell counts and viral loads in a Cote d’Ivoire cohort [14]. This study, which incorporated incremental comparisons to cheaper therapies, found ART without CD4 testing to cost US$620 per life-year gained when compared with cotrimoxazole prophylaxis, and $1180 per life-year gained if the ART initiation decision incorporated CD4 test results.

Another simulation study set in resource-poor settings estimated that ART with first-line antiretroviral regimens only would cost US$628 per QALY gained [15]. This study also estimated $238 per QALY gained for CD4 monitoring and $16,139 for viral load monitoring of ART.

A recent modelling study examined expanding ART indications to CD4 < 350 cells/mm^3^ for South Africa, examining a wide range of specific antiretroviral drug regimens [16]. They found three drug combinations to be economically efficient at a CD4 count below 350: stavudine one regimen available ($610 per year of life saved [YLS]), tenofovir one regimen (US$1140 per YLS), and tenofovir two regimens available (US$2,370 per YLS). This analysis did not consider prevention benefits.

Finally, one of us co-led a CEA of home-based ART in Uganda which included benefits to patients, family members, and uninfected sexual partners [17]. This study used a computer-based, deterministic cost-effectiveness model to assess cost-effectiveness of HAART and cotrimoxazole prophylaxis *versus* cotrimoxazole alone, and with the period before either intervention. Data for two years were derived from a trial of HAART in 1045 patients in Tororo District in eastern Uganda. Costs and outcomes were projected out to 15 years. First-line HAART regimen consisted of standard doses of stavudine, lamivudine, and either nevirapine or, for clients with active tuberculosis, efavirenz. Second-line therapy consisted of tenofovir, didanosine, and lopinavir/ritonavir.

The HAART programme standardised for 1000 patients cost an incremental US$1.39 million in its first two years. Compared with cotrimoxazole prophylaxis alone, the programme reduced mortality by 87%, and averted 6861 DALYs. Benefits accrued from reduced mortality in HIV-infected adults (67.5% of all benefits), prevention of death in HIV-negative children (20.7%), averted HIV infections in adults (9.1%) and children (1.0%), and improved health status (1.7%). The net programme cost, including the medical cost implications of these health benefits, was $4.09 million. The net cost per DALY averted was US$597 compared with cotrimoxazole alone.

## COST-EFFECTIVENESS OF HIV PREVENTION

There is an oft-repeated refrain in discussions of health care funding that prevention saves money, and for this reason (as well as health benefits) it should be well funded. Unfortunately, this broad characterisation does not withstand scrutiny: prevention often provides good value for money (e.g. a low cost per DALY averted), but rarely results in net savings. HIV prevention is the most prominent exception: many HIV prevention interventions yield net savings. That is, they cost less per HIV infection averted than the lifetime cost of treating an HIV infection with ART (estimated US$6000 - $12,000 in the developing world, half as much without ART; working paper). Thus, for HIV prevention, to compare interventions it is most useful to look at the cost per HIV infection averted (HIA), unadjusted for averted HIV care costs.

Information on HIV prevention cost-effectiveness is increasing, but is exceeded by information on the effectiveness alone or the cost-effectiveness of other interventions. The cost per HIA in developing countries has been estimated for a modest set of interventions, in some settings [13, 18-22]. The strongest evidence for effectiveness and cost-effectiveness is for blood screening, high-risk groups, male circumcision, and preventing mother-to-child transmission (PMTCT). Below are key data. Wide ranges in some estimates reflect differences in programme costs, epidemic settings, and economic methods.

Peer education for sex workers appears very cost-effective, at US$68-$79 per HIA. Mass media has been estimated at US$58 per HIA, though with uncertainty on what constitutes an effective strategy in different settings. Adult male circumcision has been estimated at US$181 to US$1500 per HIA. Blood screening costs US$75 - 1000 per HIA, and up to US$45,000 in very low HIV concentration settings. Condom distribution has been estimated at US$10-$2,188 per HIA; and voluntary counselling and testing at US$67-$482 per HIA, mostly due to risk reduction among those who test HIV positive. Treatment of other sexually transmitted infections has been estimated at US$271-$514 per HIA, although with considerable uncertainty on effectiveness after eight trials, seven of them negative. Harm reduction (needle exchange and treatment of drug dependency) costs US$97 - 564 per HIA in Eastern Europe. PMTCT costs US$20 - 6000 per HIA. Finally, school-based programmes (educational curricula) have been estimated at US$1,350 - 13,326 per HIA, with considerable uncertainty on which programme variants work and on the duration of benefit.

## POTENTIAL EPIDEMIC IMPACT OF ART

Epidemic impact is critical for assessing the cost-effectiveness of ART for prevention. The number of HIV infections averted determines the vast majority of DALYs averted in the effectiveness portion of the ICER; changes in morbidity contribute less than 5% to total DALYs. In order to highlight these epidemic issues that are central to cost-effectiveness modelling, and for completeness, we briefly review epidemic impact here. These issues are thoroughly explored by Granich *et al.* in this issue of *Current HIV Research*.

There is accumulating empirical evidence that ART reduces HIV transmission. Sexual transmission of HIV-1 is rare among persons with levels of less than 1500 copies of HIV-1 RNA/mL [23-25]. ART dramatically lowers viral load and observational studies have demonstrated its potential for prevention of HIV transmission [25-27].

The core issue in epidemic impact is: How much does ART reduce HIV transmission? Evidence suggests that the combination of viral suppression and reduction in risky behaviours may decrease transmission by 92-98%, as per below.

One study conducted in rural Uganda modelled HIV transmission to the partners of index cases treated in an ART home care programme, based on changes in plasma viral load and condom use [28]. Six months after initiation of ART, risky sexual behaviour was reduced by 70%. Median viral load among those reporting risky sex was 122,500 copies/mL at baseline and < 50 copies/mL at follow-up. Estimated risk of HIV transmission from cohort members declined by 98%.

A 2009 meta-analysis including 11 cohorts (5021 heterosexual couples) found zero risk of sexual transmission while on ART for HIV-1 RNA below 400 copies/mL (upper confidence limit of 1.27 per 100 years), and an overall 92% reduction in transmission risk per person-year for those on ART versus untreated individuals [5]. A recent study of HIV-serodiscordant heterosexual couples in Africa found the same 92% reduction in transmission if the HIV-positive partner was on ART [25]. Most recently, the *HPTN 052* randomised controlled trial was stopped due to compelling evidence that early administration of antiretroviral therapy reduces HIV transmission in discordant couples by 96% [7].

The scientific evidence also suggests a significant community-level impact of ART on HIV transmission. In British Columbia, lower HIV incidence among injecting drug users is associated with ART use and a decrease in community plasma HIV RNA concentrations [29]. There is also evidence that higher ART use is associated with decreased HIV incidence [30]. A 2004 study from Taiwan found a 53% reduction in new HIV cases associated with free access to ART [31]. A San Francisco study found that new HIV diagnoses fell by 45% between 2004 and 2008, as average HIV viral load fell by 40% [32].

Epidemic models find large potential epidemic effects with expanded ART. Early analyses suggested that rapid scale-up of conventional ART approaches could substantially reduce mortality [33] and HIV incidence [34, 35]. A recent model of the potential impact of ART, using available data and focussing on a generalised heterosexual HIV epidemic in South Africa, found that expanding access to ART for everyone at CD4 count <350 cells/mm^3^ could have a significant impact on morbidity, mortality and HIV incidence. Expanding beyond to all CD4 levels with combined prevention interventions resulted in a 95% reduction in HIV incidence in 10 years [8]. Others suggested that expanding access to ART could foster the proliferation of ARV-resistant strains [36], thus reducing the potential benefit. However, accumulating empirical evidence suggests that these concerns are not borne out with ART in community practice, where little clinical relevant resistance has been documented (e.g. Vancouver [37]), perhaps due to the recent advances in ART that increase adherence and decrease resistance [38].

Given the compelling evidence for the transmission-suppressing effects of ART, major epidemic impact depends on delivering ART to individuals during the period when they would be transmitting HIV. This consideration is what drives the strategy to provide ART in the years when CD4 count is still high and symptoms are low. However, there is a limit to how quickly individuals can be identified and put onto ART after initial infection. Indeed, the brief initial infection period (acute phase) likely accounts for a disproportionate share of HIV transmission. Data from Rakai, Uganda suggest an 8- to 26-fold higher risk per exposure in the critical first 3 to 6 months [39, 40]. Epidemic modelling suggests that the importance of the acute phase (i.e., the contribution to total new infections) was much higher early in the epidemic, and has probably stabilised at around 30% of new infections in stable epidemics [40-42].

The importance of HIV transmission concentration in the acute phase of infection depends on the question being addressed. If the intent of an analysis is to quantify the epidemic impact of a modest level of coverage with early ART, then missing the acute phase with high transmission risk would lower potential impact and worsen cost-effectiveness, as compared with transmission risk that is evenly spread over time since infection. However, if the intent is to eliminate the epidemic, the implications are different. Due to epidemic dynamics, a high concentration of transmission in the acute phase translates to a low epidemic “reproductive rate” (R_0_) - the average number of new infections transmitted from one infected person at low HIV prevalence. R_0 _less than 1.0 means that the epidemic will die out, because HIV deaths are not fully replaced by new infections. A low R_0_ (e.g. 1.5 or 2) is implied by concentration of risk in the acute phase, because otherwise the epidemic would have grown faster than it did initially. This low R_0_ means that transmission risk needs to be lowered only a little in order to eliminate the epidemic, by suppressing new infections below replacement rate. This is encouraging for efforts to reduce HIV prevalence through high ART coverage. The details of this insight are discussed in the supplementary material to a paper by Granich *et al.* [8].

## CONCEPTUAL MODEL OF ART FOR PREVENTION COST-EFFECTIVENESS ANALYSIS

As described above, the goal of any health-related cost-effectiveness analysis is to compare the incremental cost of an intervention to its incremental gain in health (or reduction in disease burden). The metric considered here is the “incremental cost-effectiveness ratio” (ICER). The intervention is starting ART at high CD4 counts, instead of waiting for a lower CD4 count. The numerator of the ICER is the change in costs (i.e. incremental costs) associated with this intervention. The denominator is the change in disease burden.

Fully portraying the cost-effectiveness of starting ART at high CD4 counts (i.e. sooner after infection rather than later) requires examining not only prevention effects, but also clinical effects for the patient. That is, ART is not a pure prevention strategy, even at high CD4 levels; it is a clinical intervention that also provides major prevention benefits. A properly comprehensive analysis takes into account both clinical and epidemiological effects.

Fig. (**1**) illustrates the factors which affect the ICER, and which need to be accounted for in a cost-effectiveness analysis of ART at high CD4. The ICER is placed centrally. Along the top are the factors which are affected by starting ART at high CD4, and which in turn affect health care costs (the ICER numerator). Along the bottom are a nearly identical set of factors affected by early ART, and which affect disease burden (the ICER denominator).

Assessing the influence of several of these factors requires a representation of clinical progression, including disease worsening and ART clinical management issues. This can be based on empirical data, but usually also requires simulation modelling to project over several years. The estimated HIV prevention benefit (averted HIV infections) requires data and modelling of HIV transmission (i.e. epidemic dynamics).

Taken from left to right:

- ***Early start of ART*** adds costs immediately - the antiretroviral drugs, monitoring tests, staff time, and so on. Clinically, early ART likely reduces disease burden, by reducing the (relatively low) mortality at that stage of illness [43]. The inconvenience and usually minor side effects of taking ART might add slightly to disease burden. Understanding the effects of this parameter requires only empirical estimates of costs and clinical effects; modelling is not necessary.
- ***Averted morbidity*** decreases both costs and disease burden. Averted disease, e.g. HIV-related opportunistic infections, results in lower health care costs (e.g. ambulatory, drugs, inpatient). However, the decreases in disease burden are modest, since the effect on DALYs of avoiding morbidity (as opposed to mortality) is moderated by transience and only partial compromise to health.
- Portraying these effects (and the next two in this list) requires clinical modelling or very long-term clinical data, since the effects accumulate over 5-10 years, as ART slows disease progression and keeps individuals healthy over time. Proper quantitative estimation requires data on rates of morbidity by CD4 level, and the effects of ART on those rates. It also requires data on the costs of managing those disease episodes.
- Increased survival drives the next factors: ***Longer duration of ART ***and*** More use of second-line antiretroviral drugs ***both increase costs. The former is a direct function of the increased survival that derives from decreased rate of disease progression, as reflected in a decreased rate of decline in CD4. The latter is more nuanced and uncertain in magnitude. Over time, first-line ART regimens will fail, by clinical or immunologic criteria. However, the rate of failure and switch to second- and third-line regimens is better understood for populations with lower CD4 counts and at correspondingly greater risk of adverse clinical events. Nonetheless, a model must make estimates, based on available data. The higher the rate of change to second- and third-line therapies, the higher the overall antiretroviral drug costs. Some models also consider the potential rate of community antiretroviral resistance, but this concern is thus far theoretical, not borne out by empirical data [37]. On the clinical side, ***longer survival*** contributes substantially to reduced disease burden. This benefit takes years to accumulate, since individuals with high CD4 would usually survive at least 5 years without ART. Although morbidity benefits may accrue relatively quickly, averted mortality starts later. When it does occur, an averted death reduces disease burden (DALYs) far more quickly than averted morbidity. Each added year of life averts one DALY, while each morbid event averted tends to add less than one twentieth of a DALY - e.g. with illness duration of one month, at 50% disability (1/12^th^ * 0.5 = one 24^th^ of a DALY). Longer survival also increases the time during which the patient can transmit HIV infection. However, as discussed below, this effect appears to be far lower than the suppression of HIV transmission by ART.
- ***Averted HIV infections*** is the prevention effect of - and prime motivation for - starting ART at high CD4. The benefits are large, approximately seven (discounted) DALYs per HIV infection averted. The averted infections start quickly, as soon as viral load is suppressed. However, the clinical benefits are delayed, since clinical disease associated with HIV infection appears only 5 to 7 years after infection. Similarly, the “booked” averted economic costs of averting HIV infection occur fairly quickly, but the actual cost benefits associated with decreased use of health services are delayed until the time of clinical disease development.

Proper estimation of averted HIV infections requires sophisticated epidemic modelling. Typical modelling elements include HIV prevalence and incidence (ideally by age, gender, and risk group), the rate of risky sexual contacts (e.g. unprotected sex episodes per year), the risk of HIV transmission per contact, and the reduction in risk of transmission due to ART.

The value of the ICER is determined by the overall values for costs and disease burden, i.e. the aggregate net effects of the above factors. Importantly, the influences on costs are both favourable (decreased costs) and unfavourable (increased costs), whereas the influence on disease burden is almost uniformly favourable (lowered). Thus, the net effect of competing cost influences may be to raise or lower total costs. This net cost varies over time, since initial effects (such as starting ART) tend to increase costs, whereas some longer-term effects (such as averting morbidity or infections) tend to decrease costs. The net cost, and the ICER, typically evolves favourably over 5 to 10 years.

Several critical implementation issues not represented in this conceptual figure affect the achievable scale of benefit. One of these issues is ***uptake***: what portion of individuals with high CD4 will agree to start ART. With recent evidence of mortality benefits even at high CD4 [43], there is a personal motivation. However, uptake rates have varied widely by setting. Lower uptake will decrease the broad impact of an early ART strategy. It is likely to leave the ICER mainly unaffected (costs and disease burden affect scale in similar proportion). Similarly, ***retention*** in care affects the scale of benefit achievable, though less so the ICER.

A critical issue at the juncture of biology and implementation is the extent to which HIV transmission is concentrated in the*** acute phase of HIV infection***. Accumulated evidence suggests that 20 to 40% of HIV transmission may occur in the first several months of infection, due to high viral load and transmissibility, as well as potentially higher sexually activity (e.g. Powers *et al.* [44]). HIV testing programmes have a hard time finding individuals early in the acute phase, and getting them rapidly into ART. Thus, depending on the estimated concentration of transmission in the acute phase, and the timing of detection and treatment, a significant portion of potential infections averted (and associated cost and burden gains) may be missed, with little reduction of the cost of ART. In our modeling, this portion is about one third of the total. We recognise that this assessment depends on epidemiologic context and is uncertain; for example, surveillance data from South Africa are also consistent with an acute phase role below this range [8].

## ART AS PREVENTION, PRELIMINARY ESTIMATES OF COST-EFFECTIVENESS

We are aware of four cost-effectiveness analyses of initiation of ART at higher CD4 levels, considering prevention benefits. Two are for North America, one for Russia, and one, for South Africa, is our own, presented at meetings and under review at a medical journal.

One analysis focused on the potential expansion of ART in British Columbia, Canada, from current 50% to 75% coverage [45]. This study combined a mathematical model of HIV transmission with a microsimulation model describing the clinical and economic course of HIV. Direct medical costs included antiretroviral and other medications, hospitalisations, physician visits, and laboratory tests. Extensive longitudinal data were available for health services utilisation, clinical progression, and survival. All analyses employed individual-level simulations, and were parameterised based on individual-level data for all persons in British Columbia receiving treatment with HAART. CD4 and viral load trajectories after initiation of HAART were described by nonlinear statistical models, and direct medical costs by random effects models that incorporated both utilisation and level of use of particular health services. Consistent with the AIDS epidemic in British Columbia, the analysis assumed that 83% of individuals were men, 72% were injection drug users, and average medication adherence (the proportion of months receiving ART) was 79%. The analysis estimated net costs, HIV infections averted, and quality-adjusted life years (QALY). It assumed a willingness-to-pay threshold of US$50,000 per QALY.

The analysis found a cost per QALY gained of $241,000 in the first year, decreasing to $5200 over 30 years as benefits accumulate (K Johnston, personal communication). Incorporating a willingness to pay of US$50,000 per QALY, over 30 years the HAART expansion scenario was associated with a net benefit of US$ 900 million (95% confidence interval US$ 493 million to 1.45 billion). The authors concluded that increasing the ART rate from 50 to 75% of clinically eligible individuals in British Columbia is cost-effective as a public health intervention.

Another recent analysis examined the effects on the US HIV epidemic of expanded HIV screening and ART [46]. This study used a dynamic mathematical model of HIV transmission and disease progression, over 20 years. The target populations were high-risk (injection drug users and men who have sex with men) and low-risk persons aged 15 to 64 years. The analysis found that one-time HIV screening of low-risk persons coupled with annual screening of high-risk persons could prevent 6.7% of a projected 1.23 million new infections at a cost of US$22,382 per QALY gained, assuming a 20% reduction in sexual activity after screening. Expanding ART to 75% of persons eligible under current CD4 guidelines prevents 10.3% of infections and costs US$20,300 per QALY gained. A combined strategy prevents 17.3% of infections at a cost of US$21, 580 per QALY gained. With no reduction in sexual activity, expanded screening prevents 3.7% of infections. Earlier ART initiation (when CD4 count is greater than 350 cells/mm^3^) prevents 20% to 28% of infections. Additional efforts to halve high-risk behaviour could reduce infections by 65%. The authors concluded that even substantial expansion of HIV screening and treatment programmes is not sufficient to markedly reduce the US HIV epidemic without substantial reductions in risk behaviour.

An analysis set in St. Petersburg, Russia, assessed the effectiveness and cost-effectiveness of providing ART to HIV-infected injection drug users (IDUs) and non-IDUs [47]. The analysis used a dynamic HIV epidemic model which divides the adult population on the basis of injection drug use and HIV status. With no incremental ART, HIV prevalence was predicted to reach 64% among IDUs and 1.7% among non-IDUs after 20 years. With ART for IDUs, over 40,000 infections would be prevented (75% among non-IDUs), adding 650,000 QALYs at US$1501 per QALY gained. ART targeted to non-IDUs would avert fewer than 10,000 infections, adding 400,000 QALYs at US$2572 per QALY gained. Untargeted ART prevented the most infections, adding 950,000 QALYs at US$1827 per QALY gained. The authors concluded that ART in St. Petersburg, Russia would generate large health benefits and be economically efficient.

Our own analysis examines the potential treatment and prevention benefits of expanded access to HIV services including ART in South Africa. We model HIV testing at 90% annual coverage in adults 15-49 years old and four ART expansion scenarios: 1) current practice (ART for CD4 count < 200 cells/mm^3^); and ART for 2) CD4 count <350 cells/mm^3^, 3) CD4 count <500 cells/mm^3^, and 4) all CD4 levels. We portray health care infrastructure and utilisation, ambulatory and inpatient costs, and the effect of ART on costs using data primarily from South Africa. Drug costs reflect 2009 international generic antiretroviral prices. ART reduces transmission per person-year by 92%. We assume best practice ART programme functioning, and present analyses with and without enhanced prevention (assumed to lower incidence by 40%), for elevated acute phase transmission risk, and other one-way and multivariate sensitivity analyses.

We find that versus current practice, expanding ART to CD4 count <350 cells/mm^3^ is estimated to prevent about 15% of new HIV infections, deaths, and DALYs over 40 years. Costs of expansion to CD4 < 350 cells/mm^3^ are offset by lower hospital costs, allowing cost break even within two years, with savings of one half billion US dollars over 5 years and US$4 billion over 40 years. Expanding to all CD4 levels further decreases HIV infections by 45% and costs by US$10 billion over 40 years, with economic breakeven in 12 years. Expanding ART in the context of expanded traditional HIV prevention reduces ART-associated DALYs averted by 18% and savings by 15%, as compared with a “current prevention” context. With higher ART and monitoring costs, ART expansion still costs less than US$200 per DALY averted over 40 years. High ART drop-out and assuming acute phase concentration of HIV transmission reduce DALYs averted by one quarter and savings by one tenth. We conclude that increasing the provision of ART has the potential to reduce costs substantially over 5 and 40 years in South Africa, while sharply reducing the burden of HIV. HIV testing uptake and ART retention and adherence determine effectiveness, and should be evaluated in field trials.

## FUTURE DIRECTIONS

The economic evaluation of expanded ART for prevention will likely take two paths: additional modelling and analyses considering a range of contextual and biological factors, and empirical assessment of programmes operating at increasing scale. These methods will inform each other *via *a feedback loop: analyses will help shape data collection, and empirical data will help models calibrate to on-the-ground realities.

Economic analyses to date have been few, as noted above. There have been more epidemic models, without economic components. However, there is a great need for models that explore the implications of the wide range of factors that determine cost-effectiveness, that vary across settings. These include:

- *Epidemic conditions:* HIV prevalence and incidence have a large impact on the potential cost of and savings from expanded ART. Higher HIV prevalence increases the cost of expanded ART, and higher HIV incidence increases the infections averted and associated medical care cost savings. In general, the two epidemic indicators run in tandem, providing offsetting effects on net cost. However, specific epidemic situations may be more or less economically favourable for expanding ART.
- *Economic factors:* The price of inputs (labour, drugs, supplies, medical care in general, and so on) affects the level of the net savings. For example, in our South Africa analysis, the relatively high cost of hospital days, as compared with the cost of a year on ART, increased the attractiveness of expanding ART, producing net savings in several years for certain scenarios.
- *Health system capacity:* In order for ART to expand rapidly, the health system must be able to deploy resources efficiently. This may mean mainly a shift in resources from one setting (e.g. inpatient) to another (e.g. outpatient). It may also mean new and efficient delivery models - an optimal combination of facility and community-based services, a mix of public and private sector, and task shifting to lower health cadres. Regardless, it may mean, at least for a time, an increase in overall resources required.
- *Early ART programme performance:* Little is known about how well the best programmes can enroll and retain patients, and how closely large-scale programmes can approximate these best practices. Some ART programmes (e.g. Malawi) have performed remarkably well, but others have not. As data accumulate on operating programmes, models can quickly take these data into account.
- *Importance of the acute phase in HIV transmission:* As noted earlier, greater concentration of HIV transmission in the first months after infection makes an expanded ART programme initially less economically attractive. However, this high early transmission has implications for the “reproductive rate” (R_0_) of HIV, and may mean that epidemic elimination is easier to achieve - with expanded ART and other prevention strategies (e.g. male circumcision and a potential vaccine).

Empirical assessments of programmes are almost always more compelling than simulation models, since they replace input parameter value estimates and complex calculations with directly measured outcomes. Among the factors discussed above, many are potentially amenable to direct measurement: programme performance (enrollment and retention); economic factors (costs of the programme and offsetting savings from decreased health services use); and HIV incidence (in selected sex partners). Policy-makers and funders need the evidence provided by large-scale demonstration projects.

Empirical economic research plays a critical part in such field evaluations, examining:

- *Cost of ART provision:* divided into service component, examined for variation by programme setting or approach (such as task shifting - alternate use of health personnel), and taking into account programme performance (including attrition and the need for retention counselling).
- *Changes in medical care utilisation and costs:* hospital and ambulatory care use unrelated to the ART provision (e.g. for opportunistic infections) are expected to drop, and the magnitude of this drop has huge implications for the net cost of ART, as well as for resource needs overall and by sector.
- *Changes in economic productivity:* Many individuals receiving ART will have been symptomatic, despite relatively high CD4 counts, and may experience decreased disability and increased economic productivity.
  Finally, simulations and empirical data interact: economic analyses will rely on non-economic empirical outcomes. These outcomes include programme performance (e.g. coverage levels) and observed changes in HIV incidence. The economic analyses, with epidemic modelling components, will use these results to improve the cost-effectiveness estimates and insights. Cost-effectiveness analyses may be able to point to those programmatic factors which if addressed would most favourably influence the cost, effectiveness, and cost-effectiveness of ART for prevention.

## DISCLAIMER

The opinions and statements in this article are those of the authors and do not represent the official policy, endorsement or views of the World Health Organization.

## Figures and Tables

**Fig. (1):**
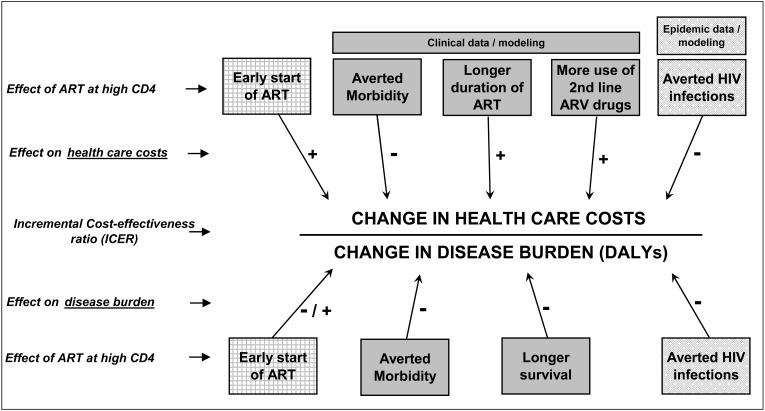
Conceptual model for assessing ART cost-effectiveness, including prevention benefits. ART, antiretroviral therapy; ARV, antiretroviral; DALY, Disability-adjusted life year.

**Table 1.** Explanation of Disability-Adjusted Life Years (DALYs) and Quality-Adjusted Life Years (QALYs)

	Geographic Setting	Measures	Components	Goal is to:	Discounting of Future Events
Disability-Adjusted Life Years (DALYs)	Global, and developing world	Disease burden	“LY” is life years lost due to premature death. “DA” is disability* due to morbidity.	Avert	Yes, 3% per year
Quality-Adjusted Life Years (QALYs)	U.S., Europe, and othe OECD countries	Health status	“LY” is gain in life years due to intervention. “QA” is gain in health status utility* due to better health.	Gain	Yes, 3% per year

* In practice, methods to estimate disability weight and health status utility often overlap, relying on similar elicitation of expert opinion. DALY disability weights by disease are available at http://www.who.int/healthinfo/global_burden_disease/2004_report_update/en/index.html.

**Table 2.** Key Types of Cost Analysis in Health

	Definition	Outcome meTric	Conceptual Origin	Use to Decide what?	AIDS Examples
**Cost**	Cost of resources required to deliver an intervention.	$ (currency)	Programme management	What level of resources are required to deliver health programmes or care?	$500 to provide anti-retroviral therapy (ART) for one year. $50 for a circumcision.
**Net cost**	Costs to deliver intervention, adjusted for offsetting savings due to disease averted.	$ (currency)	Cost-effectiveness and cost-benefit analysis	What is the net expenditure expected with delivery of health programmes or care?	$400 per year of ART, adjusted for averted opportunistic infections (but in this example not for averted HIV infections). Net savings for circumcision, adjusted for averted HIV infections and associated costs.
**Cost-effectiveness analysis (CEA) - in general**	Net cost per added unit of health	Incremental Cost Effectiveness Ratio (ICER) - net cost/net health benefit (death averted, year of life)	Decision analysis (especially in medicine & health), plus costs	What’s the incremental cost per added health benefit?	Cost per AIDS death averted. Cost per HIV infection averted.
**Cost-effectiveness analysis (CEA) - with a standardised health metric**	Net cost per added standardised unit of health (a ratio). Most common efficiency metric in health economics.	ICER (net$/QALY gained) ICER (net $/DALY averted)	Specific version of CEA-permits comparison across diseases and clinical outcomes	What’s the incremental cost per added QALY? Or per averted DALY? Is it worth it *vs* threshold? Prioritising. Per WHO, $per DALY< annual GDP * per capita* is “very cost-effective”.	Prevented HIV infection averts about 7 DALYs. A year of ART averts about 0.75 DALY. ART ICER = $600 per DALY averted (without prevention benefits). Circumcision has no ICER because it saves money.
**Resource allocation (RA)**	Assignment of available resources to different uses.	Total QALYs gained or DALYs averted for available funds	Budgeting exercises - government organisations	How to spend a limited budget. Proceed from low to high cost-per-QALY interventions.	How do we divide funds between prevention and treatment? Between ART and CD4 monitoring?
**Cost-benefit analysis (CBA)**	Cost of intervention minus savings (a difference, not a ratio) Less often used in health economics.	Net $ (B - C) *Typically includes benefits beyond health*	Welfare economics	Is the programme worth doing? i.e. produce more value than it costs?	ART may save money due to prevented HIV infections, as well as increased economic productivity of PLWH and family.

QALY = quality adjusted life year, a measure of health that tallies years alive adjusted for health status of those years. Cost per QALY gained often called cost-utility analysis. DALY = disability adjusted life year, a measure of disease burden that sums premature mortality (LY) plus disabling morbidity (DA). WHO = World Health Organization; GDP = Gross Domestic Product; PLWH = Person Living with HIV.

## ABBREVIATIONS

AIDS: = Acquired Immune Deficiency Syndrome
ART: = Antiretroviral Therapy
ARV: = Antiretroviral
CA: = California
CBA: = Cost-Benefit Analysis
CE: = Cost-Effectiveness
CEA: = Cost-Effectiveness Analysis
DALY: = Disability-Adjusted Life Year
GDP: = Gross Domestic Product
HAART: = Highly Active Antiretroviral Therapy
HIA: = HIV Infection Averted
HIV: = Human Immunodeficiency Virus
HPTN: = HIV Prevention Trials Network
ICER: = Incremental Cost-Effectiveness Ratio
IDU: = Injection Drug User(s)
OECD: = Organisation for Economic Co-operation and Development
PEPFAR: = United States President’s Emergency Plan for AIDS Relief
PLWH: = People Living With HIV
PMTCT: = Prevention of Mother-to-Child Transmission
QALY: = Quality-Adjusted Life Year
R_0_: = Reproductive Rate
RA: = Resource Allocation
UCSF: = University of California San Francisco
UK: = United Kingdom
US(A): = United States (of America)
WHO: = World Health Organization
YLS: = Year of Life Saved
